# Experimental Investigation of the Atmosphere-Regolith Water Cycle on Present-Day Mars

**DOI:** 10.3390/s21217421

**Published:** 2021-11-08

**Authors:** Abhilash Vakkada Ramachandran, María-Paz Zorzano, Javier Martín-Torres

**Affiliations:** 1 Group of Atmospheric Science, Department of Computer Science, Electrical and Space Engineering, Luleå University of Technology, 97187 Luleå, Sweden; 2 Centro de Astrobiología (CSIC-INTA), Torrejón de Ardoz, 28850 Madrid, Spain; zorzanomm@cab.inta-csic.es; 3 School of Geosciences, University of Aberdeen, Aberdeen AB24 3FX, UK; javier.martin-torres@abdn.ac.uk; 4 Instituto Andaluz de Ciencias de la Tierra (CSIC-UGR), 18100 Granada, Spain

**Keywords:** Mars, pure liquid water, water cycle simulation, habitability, planetary protection, ISRU

## Abstract

The water content of the upper layers of the surface of Mars is not yet quantified. Laboratory simulations are the only feasible way to investigate this in a controlled way on Earth, and then compare it with remote and in situ observations of spacecrafts on Mars. Describing the processes that may induce changes in the water content of the surface is critical to determine the present-day habitability of the Martian surface, to understand the atmospheric water cycle, and to estimate the efficiency of future water extraction procedures from the regolith for In Situ Resource Utilization (ISRU). This paper illustrates the application of the SpaceQ facility to simulate the near-surface water cycle under Martian conditions. Rover Environmental Monitoring Station (REMS) observations at Gale crater show a non-equilibrium situation in the atmospheric H_2_O volume mixing ratio (VMR) at night-time, and there is a decrease in the atmospheric water content by up to 15 g/m^2^ within a few hours. This reduction suggests that the ground may act at night as a cold sink scavenging atmospheric water. Here, we use an experimental approach to investigate the thermodynamic and kinetics of water exchange between the atmosphere, a non-porous surface (LN_2_-chilled metal), various salts, Martian regolith simulant, and mixtures of salts and simulant within an environment which is close to saturation. We have conducted three experiments: the stability of pure liquid water around the vicinity of the triple point is studied in experiment 1, as well as observing the interchange of water between the atmosphere and the salts when the surface is saturated; in experiment 2, the salts were mixed with Mojave Martian Simulant (MMS) to observe changes in the texture of the regolith caused by the interaction with hydrates and liquid brines, and to quantify the potential of the Martian regolith to absorb and retain water; and experiment 3 investigates the evaporation of pure liquid water away from the triple point temperature when both the air and ground are at the same temperature and the relative humidity is near saturation. We show experimentally that frost can form spontaneously on a surface when saturation is reached and that, when the temperature is above 273.15 K (0 °C), this frost can transform into liquid water, which can persist for up to 3.5 to 4.5 h at Martian surface conditions. For comparison, we study the behavior of certain deliquescent salts that exist on the Martian surface, which can increase their mass between 32% and 85% by absorption of atmospheric water within a few hours. A mixture of these salts in a 10% concentration with simulant produces an aggregated granular structure with a water gain of approximately 18- to 50-wt%. Up to 53% of the atmospheric water was captured by the simulated ground, as pure liquid water, hydrate, or brine.

## 1. Introduction

Liquid water is a requirement for life as we know it. The triple point of water is at 273.16 K (0.01 °C; 32.02 °F) and a partial vapor pressure of 6.1166 mbar (611.66 pascals). The atmosphere of Mars is generally dry, and, although the surface pressure may be above this point, the partial vapor pressure is generally much lower and therefore, liquid water is not stable on the surface of Mars. However, the recent measurements of relative humidity (RH) provided by the Rover Environmental and Monitoring Station (REMS) onboard the Curiosity rover have shown that saturation may be reached at the surface at night, even at near-equatorial latitudes [1]. Previous landed missions have demonstrated it visually. In particular, in 1977, a thin layer of frost was seen at the Viking Lander 2 (VL2) landing site at Utopia Planitia (47.64° N 225.71° W). The first time occurred in 1977 [2] and then it was spotted again, one Martian year later, in 1979 [3], and both times during the northern hemisphere winter. Levin and Levin, 1998 [4], suggested that the ground may act at night as a cold sink scavenging atmospheric water, trapping all the available moisture in the total column of air, and then, when the temperature increases during sunrise, the soil may retain minute quantities of liquid water while it evaporates. Recent theoretical studies have investigated this process at the VL1 and VL2 sites, using a column model (with cloud–radiation interaction and Prandtl slope wind terms), which had been validated before with the Phoenix and Curiosity in situ observations [5]. The model predicts diffusion and adsorption of atmospheric water into the regolith in the evening, including the formation of very thin layers of frost on the ground from about midnight and an early morning fog when this water is released (i.e., a saturated air up to 1.6 m above the surface). The theoretical model also explains why frosts sublimate after sunrise, allowing desorption and diffusion of water off the regolith.

In 2002, Hecht investigated theoretically and experimentally the metastability of liquid water on Mars [6]. In the experiments, he monitored the evaporation of water ice with an atmosphere of N_2_ at room temperatures and low pressures with the water ice at temperatures between 0 and 10 °C. The measured evaporation rates were successfully compared with the theoretical predictions. In particular, he obtained a surface evaporation rate of 0.055 g/cm^2^/h at 6.7 mbar. Later on, the experimental results from [7], using CO_2_ and lower temperatures, calculated an evaporation rate of 0.023 g/cm^2^/h. These experiments suggested that thin films of liquid water may be transiently stable when ice (or frost) evaporates.

The recent observations of REMS in the Curiosity rover at Gale crater have shown strong thermal gradients between the surface and the air above Mars. In particular, the REMS instrument typically measures a ground temperature of 180 K at night and at 1.6 m above the surface, an air temperature of 200 K [1]. This temperature difference produces a gradient in water saturation vapor density, leading to a gradient in vapor pressure that produces thermodynamic non-equilibrium situations. This large diurnal thermal contrast is characteristic of Mars at Gale and other sites, such as those explored by the Phoenix mission [8] and InSight [9]. At night, the surface may become saturated and form frost or be absorbed by the surface, at least on a small scale, due to the drastic temperature drop. Then with the increase in temperature during the day, this water may be released back to the atmosphere. In this work, we shall simulate experimentally this trend allowing for the temperature to go above the triple point temperature while monitoring simultaneously in real-time the changes in relative humidity and water vapor pressure that occur within the air above the ground while these changes take place in a situation that is comparable to the one observed at Gale. To our knowledge, this is the first time that this monitoring has been performed.

Recent research has focused on the interaction of salts in the soils with atmospheric water. There have been numerous studies that suggest that sulphates, perchlorate and chloride salts in the regolith can absorb water from the atmosphere, forming hydrates, by absorption, and then liquid brines, through deliquescence and hydration [10,11,12,13,14,15,16,17,18,19,20,21,22]. Brines are aqueous saline solutions where the presence of salts decreases the freezing temperature of the solution and its saturation water vapor pressure [23], allowing it to capture water from the atmosphere when the relative humidity (RH) is higher than the threshold, which is known as deliquescence relative humidity (DRH) [24]. It has been speculated that the salts present in the Martian regolith can absorb atmospheric water and produce certain features at a large scale. Recurring slope lineae (RSL), a geomorphological feature observed on Mars, are suggested to have been induced due to the brine activity [25]. A recent theoretical model by [26] investigated the properties of theoretically (meta)stable brines at equilibrium with the ambient atmosphere and found that brines can develop and persist over 40% of the Martian surface and occur transiently for around 3 h per sol, which is 0.4% of the Martian year during the summer from the equator to high latitudes. Contrary to the case of frost formation, the absorption of atmospheric water by salts does not require RH = 100%, i.e., saturation conditions. If salts exist in the regolith, they will absorb atmospheric moisture and saturation (and thus frost formation) is less probable.

There are also previous studies that have focused on characterizing the atmosphere–regolith interchange [27,28,29,30,31,32,33]; and several past experimental studies have focused on surface morphology changes produced by water under Mars-like conditions [6,31,34]. Also [35] has studied the effect of wind and temperature on the sublimation rate of pure water ice under Mars conditions and concluded that temperature is the most critical parameter that controls the sublimation rate of water.

In this work, we demonstrate experimentally (and visually) the stability of thin films around the triple point of water. We simulate an environment that mimics the night-to-day thermal transition, and we will compare it with the observed changes in water vapor partial pressure at Gale crater, Mars. We compare the measured evaporation rates and quantify the duration of the metastability of this pure liquid water, which may be available for life. For comparison, we have also studied the behavior of certain deliquescent salts as pure components or mixed in the regolith to demonstrate the timescale of absorption under Martian experimental conditions. The future ExoMars 2022 mission will conduct the HabitAbility: Brines, Irradiance and Temperature (HABIT) experiment, which will be devoted, among other things, to investigating this interaction of deliquescent salts with atmospheric moisture in situ [36].

The purpose of this work is two-fold: (1) to investigate the stability of pure water under Martian conditions in the vicinity of the triple point, and (2) to quantify the maximal absorption capability of salts and regolith by absorption of atmospheric moisture. This work has implications for the definition of Special Regions in planetary protection, developed to protect the regions where there is water ice in the near-surface [37]. If liquid water can be transiently stable during the night-to-day transition, all locations which eventually reach saturation conditions at their surface may need to be incorporated into this category.

## 2. Diurnal Water Cycle at Gale Crater

After more than 4.5 Martian years of operations, the rover Curiosity has acquired an unprecedented data record of near-surface measurements that provides invaluable ground truth to the moisture content of the lower atmosphere [1,38]. The coordinated measurements of REMS and DAN (Dynamic Albedo of Neutrons, which monitors the subsurface H- content) onboard the Curiosity rover, along its traverse, have suggested that there is a water interchange between the soil and the atmosphere that changes seasonally [1]. Some sensors have been carefully recalibrated during operations [39], and all the data published in the Planetary Data System (pds.nasa.gov, accessed on 8 November 2021) have been reviewed and updated accordingly. We have analyzed here the diurnal and seasonal water cycle using REMS and Curiosity data throughout 2027 sols (almost three Martian years) corresponding to a traverse of the rover on Mars of 18,790 m after 2456 drive maneuvers.

**Figure 1:**
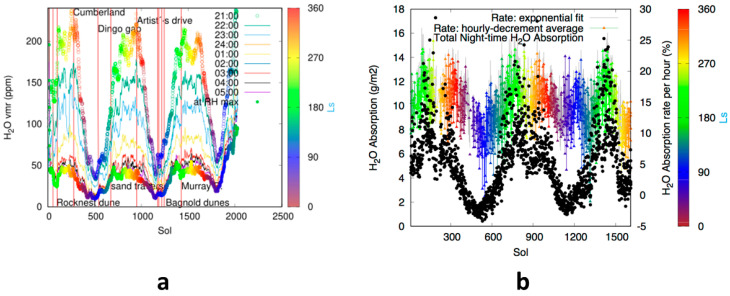
Night-time water behavior inferred at Gale crater from REMS data. (**a**) Night-time water VMR (units of ppm) for the first 2027 sols of Curiosity operations. The different colored lines show the maximum value within each hour interval as measured from 21:00 (to 22:00) to 4:00 (to 5:00) local time. The time of the year (Ls) for a given measurement is shown using the color scale on the right. (**b**) Night-time water absorption rates for the first 2027 sols of Curiosity operations. The rate calculated with the exponential law is coincident with the hourly variation. The measured night-time decrement of VMR between 21:00 and 05:00 LMST is converted into g/m^2^ for each sol of the mission (here at 230 K mean temperature and scaled for 6.1 mbar).

Figure 1 shows the maximum volume mixing ratio (VMR) values at the time of REMS measurement for every night, between 21:00–05:00 Local Mean Solar Time (LMST), during the mission. Figure 1 uses reliable data from REMS corresponding to local times between 21:00 and 05:00 (labelled as 04:00 for the maximum in the 04:00 to 05:00 period, with equivalent notation for other hours). The solar longitude (Ls) is marked in color. Figure 1a,b show a non-equilibrium situation in the atmospheric H_2_O VMR, that at Gale only occurs at night-time, as inferred from REMS data. The amount of atmospheric H_2_O decreases at night, from 21:00 through 05:00, and is sensitive to the place where the rover is standing (see Figure 1a). Some of the sites show a strong and differentiated behavior, with a much more substantial reduction than other nearby sites visited in the same season, suggesting again the existence of an atmospheric–regolith water interchange mediated by the regolith (Figure 1a). This analysis shows that the atmospheric conditions during the night change, and it looks as though there was a large sink of water in the soil whose behavior changes with the temperature of the ground. Indeed, during these hours the ground temperature drops, reaching its minimal value at about 5:00. The measured night-time decrement of VMR between 21:00 LMST and 05:00 LMST is converted into g/m^2^ for each sol of the mission in Figure 1b. This image also includes the water absorption rate per hour, in %. There is a certain seasonality but overall, the soil can absorb up to 15 g/m^2^ within one night. This water is then released back to the atmosphere, but the daytime REMS Relative Humidity (RH) measurements are nearly zero and are therefore not reliable enough to monitor the diurnal changes of water VMR.

REMS measures the RH of the air, the ambient pressure P, the air, and ground temperatures. To calculate the VMR and later on for the next experimental sections, we use these formulae [1]:

(1) $$\mathrm{RH}\left(T\right)=\frac{P}{{\mathrm{ew}}_{\left(T\right)}}\times \frac{\mathrm{vmr}}{1+\mathrm{vmr}}\times 100$$

(2) $${\mathrm{ew}}_{\mathrm{liq}\left(T\right)}=6.112\times \exp\left(17.62\times \frac{T-273.14159}{243.12+\left(T-273.14159\right)}\right)$$

(3) $${\mathrm{ew}}_{\mathrm{ice}\left(T\right)}=6.112\times \exp\left(22.5\times 1-\frac{273.14159}{T}\right)$$

(4) $$W=\frac{M_{w\;}}{M_{d}}\times \frac{\mathrm{RH}}{100}\times \frac{{\mathrm{ew}}_{\left(T\right)}}{\left(P-\frac{\mathrm{RH}}{100}\times {\mathrm{ew}}_{\left(T\right)}\right)\times 1000}$$

(5) $$\mathrm{vmr}=\frac{M_{d}}{M_{w}\times W\times 1000}$$

where

a: air and g: ground.
RH_a_ (T_a_) can be applied to the air to retrieve vmr and then be used to calculate RH_g_ (T_g_). Here RH^i^ represents the relative humidity with respect to ice, whereas RH^l^ represents the relative humidity with respect to liquid.
P: Pressure in mbar.
vmr: volume mixing ratio in parts per million.
T_g_: Table temperature in K.
${\mathrm{ew}}_{\mathrm{liq}\left(T_{g}\right)}$: saturation partial pressure over liquid water at a given temperature.
${\mathrm{ew}}_{\mathrm{ice}\left(T_{g}\right)}$: saturation partial pressure over ice at a given temperature.
M_w_ = 18.0160 (molecular weight of water).
M_d_ = 43.3400 (molecular weight of dry air on Mars).
W = water mass mixing ratio.

Another instrument onboard the Curiosity rover, ChemCam, has measured during daytime (from 10:00 to 14:00 Local True Solar Time (LTST)) seasonal variations between two precipitable microns (pr-µm) and 12 pr-µm [38], notice that 1 pr-µm = 1 g/m^2^. The values are comparable with the REMS data shown in Figure 1b.

To investigate the impact of this cycle on the surface, we will next perform experiments where we inject a total of about 8 g of water into a chamber at Martian pressure conditions with a base surface of 30 cm × 30 cm, allowing for a total column of water of 89 g/m^2^. This corresponds to about 90 pr-µm, which is only about five times greater than the maximal observed night-time water absorption change at Gale, a dry region near the equator.

## 3. Materials and Methods

### 3.1. SpaceQ Chamber

**Figure 2:**
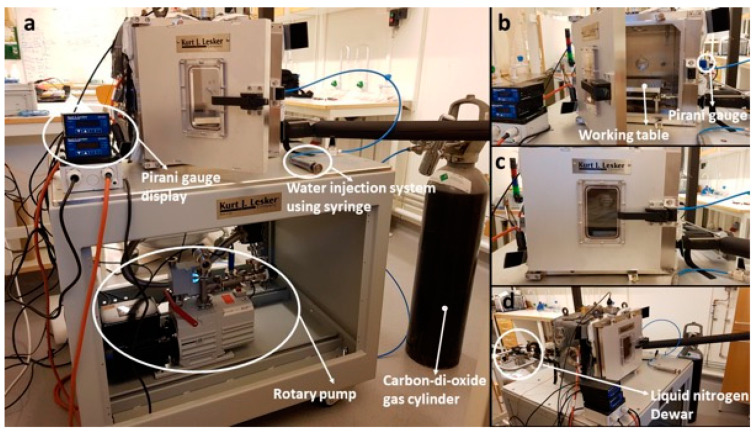
Overview of the SpaceQ chamber. (**a**) This figure shows the entire setup of the chamber, including the external Pirani gauge display, the rotary pump, the CO_2_ gas cylinder (to insert a Mars-like atmosphere) and the syringe for water injection. (**b**) Detail of the experimental table where samples are cooled down to the desired temperature and the KJLC^®^ Cold Cathode/Pirani Combination Gauge on the right side. (**c**) Viewport at the front of the chamber, used to take pictures during the experiment. (**d**) LN_2_ Dewar used to cool down the working table.

The design and development of Mars simulation facilities is still active in experimental research [40,41]. For this purpose we have developed “the SpaceQ” experimental facility [42,43], see Figure 2, to simulate the space and Mars environment for different research and technological applications. It can cover pressures from ambient to <10^−5^ mbar, and temperatures from 163 K to 423 K by (i) cooling (using liquid nitrogen, LN_2_) the plate; and (ii) heating the walls (using an external heating jacket). To measure the atmospheric temperature and relative humidity inside the chamber, and to use the heritage from the REMS RH sensor, we use a Vaisala HMT 334 sensor fitted at 10.2 cm above the plate and 5.2 cm from the sidewalls, which can work under vacuum and Martian conditions to perform water vapor studies [44]. It can measure the temperature in the range of 203.15 K to 453.15 K with an accuracy of ±2 K and the relative humidity from 0–100% RH with an accuracy of ±1% RH. This is fitted on the chamber through an M22 × 1.5 thread with the probe exposed to the inner atmosphere. The thread has been tested for vacuum-tight installations. Water is injected into the chamber using a KD Scientific stainless-steel syringe from Fischer Scientific (Figure 2a). It has a capacity of 20 mL and is fitted on to a Swagelok ¼″ connector on the right side of the chamber, which is in turn connected through a tube to a manually operated ¼″ Swagelok fitting ball valve. The water can be injected several times during the experiment, as it is communicated with the chamber through a valve. When the valve is opened, the water is injected into the depressurized environment of the chamber.

The SpaceQ chamber is a cubical facility made of stainless steel with an aluminium door and has an internal volume of 27,000 cm^3^ (27 L). The dimensions of the working table plate are 20 cm × 20 cm × 1.8 cm. The chamber consists of various optical and electrical feed-throughs with two quartz window viewports, two thermocouple feed-throughs, a vacuum compatible UV lamp, a cold cathode/Pirani combination gauge to measure the pressure, two gas inlets, ports for a vacuum pump and connections of USB, DB25 to read the data in real-time and connectors for the optical cable to spectrometer. There is also an extra port that can be used to install additional sensors if required. The pressure in the chamber is controlled by a cold cathode Pirani gauge (Figure 2b) (KJLC^®^, Liverpool UK, Cold Cathode/Pirani Combination Gauge), which has been designed for the vacuum measurement of gases in the range of 1 × 10^−9^ mbar to 1000 mbar. There are two steps involved in reducing the pressures down to 10^−5^ mbar, first using the Pfeiffer 1-phase DUO 10 M rotary pump (Figure 2a) to achieve a vacuum level of ~10^−3^ mbar, then we achieve higher orders of vacuum by coupling it with a turbo molecular pump (Pfeiffer HiPace 80). The working table is fitted with a Liquid Nitrogen (LN_2_) feed-through, which is connected to a 50 L Dewar (Figure 2d) controlled by a solenoid valve that is used to regulate the flow of the LN_2_ supply. The temperature sensor fitted on the working table is used to provide input to the thermal control unit, which acts as feedback to cool down the table to the targeted temperature. The quartz silica viewports (Zero-Length,4-1/2″ UHV) fitted on the top and left-hand side allow us to monitor the experiment or initiate an action using a specific wavelength onto the sample. The window is made up of Kodial glass (Alkali Borosilicate 7056) with a viewing area of 65 mm.

### 3.2. Salts and Mars Regolith Simulant

Calcium chloride (CaCl_2_) (anhydrous, 793,639), ferric-sulphate Fe_2_(SO_4_)_3_ (hydrate, 307,718), magnesium-perchlorate Mg(ClO_4_)_2_ (ACS reagent, 222,283), sodium-perchlorate (NaClO_4_) (ACS reagent, ≥98.0%, 410,241) are purchased from Sigma Aldrich, Stockholm, Sweden. To mimic the actual experiments of the HABIT instrument of the ExoMars platform, we also use for some tests a Superabsorbent Polymer (SAP), a Poly (Acrylamide-co-Acrylic Acid) (C_6_H_8_KNO_3_) (432,776) polymer acquired from Sigma Aldrich. The water (W4502) used for injection is from Sigma Aldrich, filtered at 0.1 µm to guarantee sterility and cleanness.

In addition to these salts and SAP we use Mojave Martian Simulant (MMS-1) as a Mars regolith simulant. These simulants consist of silica (49%), aluminum oxide (17%), iron oxide (11%), calcium oxide (10%) and small amounts of magnesium oxide and sulfates (Peters et al., 2008). They are available in different grades. We use the “fine” grade bought from The Martian Garden, Austin, Texas. There are different simulants available, such as Johnson Space Centre JSC Mars-1 [45], Salten Skov I [46], Engineering Soil (ES-x) [47], and Jining Martian Soil Simulant (JMSS-1) [48], which are used by scientists to replicate specific characteristics of the Martian regolith. A new Martian global simulant (MGS-1) has recently been developed by CLASS Exolith Lab which has characteristics of Rocknest soil at Gale crater [49] and may be used in our future studies. The Mojave Martian Simulant (MMS-1) was chosen as a regolith simulant for this study due to its availability at the time of the experiments and previous studies related to the water cycle. This simulant has been used before as an analogue for other experiments to investigate the role of dust on heterogeneous nucleation and water ice cloud formation [50].

The experiment materials are placed in glass beakers and covered with a High-Efficiency Particulate Air (HEPA) filter. This configuration mimics the configuration of the HABIT instrument of the ExoMars mission, which will carry a set of salts that will be in contact with the atmosphere and absorb water. This absorption will be indirectly monitored by measuring the electric conductivity of the resulting salt hydrate or brine [36,51].

The weight was measured with a high precision scale before and after every experiment in an ambient laboratory environment to evaluate the increase in mass that is produced by the water capturing process. A series of images and the time stamp were taken during the experiment through the viewport on the chamber and before and after the experiment.

### 3.3. Experimental Procedure

A Martian atmosphere is created by evacuating the SpaceQ chamber with a rotary pump down to 10^−3^ mbar and filling it with pure carbon dioxide (CO_2_) gas up to pressures between 5 and 8 mbar. After stabilization, water is injected at ambient temperatures using the Swagelok stainless steel syringe. Unavoidable thermal gradients occur when the table is refrigerated, and the external walls of the chamber are warmer as it is in contact with ambient laboratory temperatures. This whole process of achieving close to Martian condition takes about an hour. When water is injected, the pressure is maintained by manipulating the pressure valve of the rotary pump. The relative humidity (RH) of the air is monitored from the start of the experiment. The working table that serves as a ground surface is then cooled with liquid nitrogen (LN_2_). The table acts as a sink either forming frost or absorbing water to the salts–regolith mixtures.

**Figure 3:**
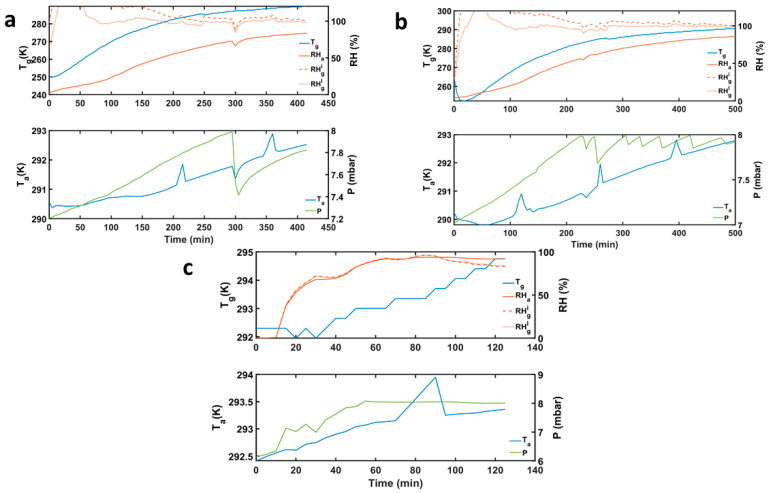
Evolution of the environmental variables inside the experimental chamber. The ground temperature (T_g_) and air temperature (T_a_), relative humidity of the air (RH_a_) and total pressure (P) are measured, whereas the relative humidity with respect to ice and liquid (RH_g_^i^, RH_g_^l^) are derived using Formulae (1)–(5). (**a**) Experiment 1: The water was injected; the table was cooled down to 250 K, and the pressure was controlled at Martian surface values. Then the temperature was raised slowly up to ambient conditions. RH_a_ and RH_g_ were significantly different due to the thermal gradient between the air and the ground. At the ground (table), the conditions are such that ice (frost) can be formed. (**b**) Experiment 2: Same conditions as in Experiment 1, with different products. (**c**) Experiment 3: Ambient temperatures.

The experiments were run over 8 to 9 h, and the evolution of the environmental variables (RH, T, and P) in every experiment was recorded once the Martian conditions were reached, as shown in Figure 3. In this set of images, the temperature of the table is represented by T_g_ to simulate the temperature of the ground surface on Mars. The temperature of the air in the chamber at the place where the RH sensor is located is indicated by Ta. The RH measured by the Vaisala probe gives the RH of the air (RH_a_), while the RH of the “ground” (RH_g_) is derived through the measured values of P, RH_a_ and Ta using the formulae given above. Experiments 1 and 2 have been run with the working table refrigerated to 250 K, to mimic the ground and serve as a cold trap.

All the experiments were run with four samples inside the chamber. In Experiment number 3, all the cycle was run with the experimental table at ambient temperatures in the range 292–295 K. After water injection, the RH reached almost saturation and the pressure increased up to 8 mbar.

For all the experiments, after the products were put in a beaker and placed onto the working table, the chamber was closed and vacuumed from 960 mbar down to close to 10^−3^ mbar. Then it was flushed with CO_2_ gas, and then CO_2_ was injected into the chamber until the atmosphere inside reached about 7 mbar. The Martian surface is simulated by a refrigerated table whose temperature is reduced with liquid nitrogen. At the end of the experiments, the atmospheric mixture of CO_2_ and water, H_2_O, was released by opening the valve, the chamber was set again to ambient atmospheric pressures. The products were extracted and weighed immediately after opening the chamber door at ambient laboratory conditions.

In Experiment numbers 1 and 2, we cool the experimental table to about 250 K because we want to study the frost formation and the occurrence of water droplets around the triple point of water. However, the air above was warmer. This situation is similar to the present-day Mars environment. At night-time, there are differences of about 20 K between the ground temperature and the air temperature where the RH sensor is, at 1.6 m above the ground [52]. When the table is refrigerated, and saturation (and over-saturation) is reached at the table (ground), the water from the atmosphere condensates rapidly on the surface, which acts as a cold trap and produces a drastic reduction of the atmospheric relative humidity. As on Mars, RH_a_ and RH_g_ have been demonstrated to be significantly different due to the thermal gradient between the air and the ground. As we shall show, at the ground (table), conditions are met for ice formation and liquid water formation. As the temperature of this plate increased slowly, this water was later released back to the atmosphere, allowing for the air RH_a_ to increase again. The relative humidity of the ground (RH_g_) is calculated using the known environmental parameters (RH_a_, T and P) and the water mass mixing ratio with Equations (1)–(5).

## 4. Results

### 4.1. Experiment 1

**Figure 4:**
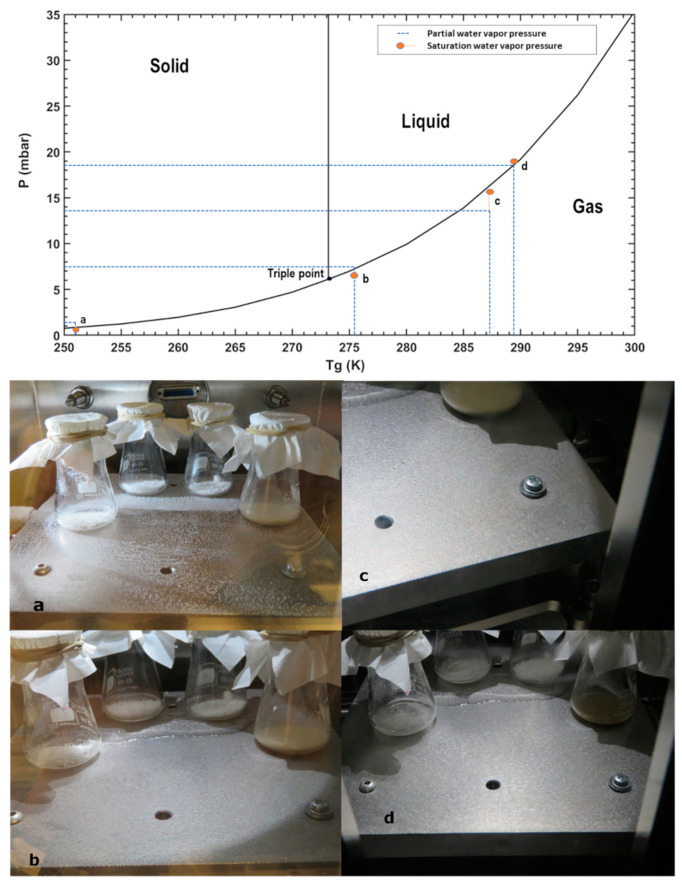
(**Top**) Derived partial water vapor pressure, T_g_ conditions and saturation water vapor pressure ${\mathrm{ew}}_{\left(T_{g}\right)}$ for liquid or ice. The surface RH_g_ was always close to 100% during this experiment, and the air was still drier. (**Bottom**) The series of images taken during the experiment shows first the formation of frost, then liquid water droplets, and a meandering narrow water flow that prevails for longer than the droplets. (**a**) As the water is added and the table temperature was lowered to 250 K, it starts to form frost. (**b**) With a gradual increase in the temperature as it passes through the triple point of water, the stable droplets appear on the table. (**c**) Then, as the temperature T_g_ increases to 287 K, the droplets start to disappear on the sides of the table. (**d**) Finally, most of the droplets evaporated as the table reached 289 K.

This experiment was conducted to study the stability of pure liquid water around the vicinity of the triple point and to observe the interchange of water between the atmosphere and the salts when the surface is saturated. For this experiment, as shown in Figure 4a, the four sample beakers of salts and SAP with 1.5 g and 0.75 g, respectively (2:1 ratio), were placed inside the chamber. At Martian pressures, we injected water using the syringe into the chamber once the air temperature stabilized. The water was injected in amounts of 0.5 mL. After the first injection, the relative humidity of air (RH_a_) increased to 2.5% and then subsequently, the water was injected 16 times as the RH_a_ reached 81%. A total amount of 8 mL (8 g) of pure water was injected. As in previous experiments [19,53], when water vapor is released from the table, which acts as a cold trap, to the air above, the pressure was maintained within 7–8 mbar by pumping the chamber (see Figure 3a). The amount of water injected was five times greater than the one observed at Gale by REMS, which is high enough to allow all the sinks (regolith or salts and solid table) to extract, absorb and retain water as needed.

As we circulated LN_2_ through the circuit that refrigerated the table that simulates the Martian surface, its temperature was reduced drastically, and the surface reached over-saturation conditions. Because of this, some water droplets appeared, and when the table temperature, T_g_, was below 250.15 K, frost started to form on the table (Figure 4a), and the cooling was stopped. While this happened, the relative humidity of the air dropped; this is due to the water absorption on the table. The refrigeration was switched off, and the table temperature was let to increase slowly to ambient temperatures. During all the execution of the experiment, the surface RH was saturated (i.e., close to 100%). As the temperature increased, water droplets started to appear at 145th min (Figure 4b). The environmental conditions inside the chamber were: table temperature T_g_ at 275.4 K, total pressure at 7.544 mbar, air temperature T_a_ at 290.76 K and relative humidity RH_a_ at 36.76%. When the temperature T_g_ reached 287.4 K with pressure at 7.523 mbar and relative humidity RH_a_ at 65.69 %, the water droplets started to evaporate (Figure 4c). After 400 min, when the table temperature T_g_ was at 289.5 K and pressure at 7.8 mbar, almost all the droplets had evaporated. At this moment, the air temperature T_a_ was 292.44 K and the RH_a_ 82.72% (Figure 4d). At the end of the experiment, the chamber door was opened, the remaining pure liquid water was absorbed with a syringe and weighed on a petri dish at ambient laboratory conditions. After 3.5 h of exposure to (P, T_g_) at the limit of the liquid–gas region of stability limit, the total amount of water collected as pure liquid water droplets from the experimental table was 0.73 g.

The partial pressures of water for each temperature, pressure, and RH measurement are shown in Figure 4 (Top) for each representative stage. As it can be seen, the pairs of table temperature (T_g_) and derived (from the measurements) partial water vapor pressures evolve exactly along the phase state boundaries. First, when frost forms on the surface by condensation of atmospheric vapor (a), the conditions lie within the boundary between solid and gaseous phases. A thin film of liquid water forms as the temperature increases and the partial pressures are just above the triple point. (b). We measured the size of the thin layer that was formed (length = 20 cm and width = 2 cm). Assuming that this film contains almost all the water, then there was a surface water density of about 0.2 g/cm^2^. This value will be used later to calculate the evaporation rate of thin films under Martian surface conditions. As the temperature continues to increase, the partial pressure always lies within the liquid and gas phases boundary, while the thin film of liquid water continues to evaporate.

**Figure 5:**
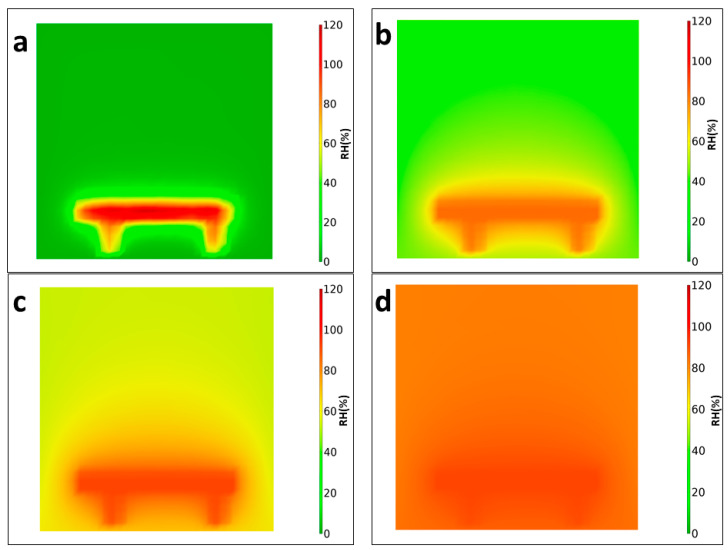
2D cross-section of the computer simulation of the relative humidity evolution (**a**) shows over saturation as the frost starts to form at the working table at 0 min. (**b**) Relative humidity of air increases as the frost sublimate to liquid water droplets at 135 min. (**c**) At 200 min increase in air relative humidity as the water is released into the air. (**d**) The relative humidity inside the chamber is uniform by the end of the experiment at 400 min.

Due to the strong thermal gradients, there are large relative humidity differences between the cold table that emulates the ground and the air above. We performed a computer simulation using COMSOL Multiphysics heat transfer to illustrate the thermal and relative humidity gradients, see Figure 5 [54]. The initial condition of the computer model considered an external boundary of the chamber at room temperature of 295 K and the working table at 250 K as in the experiment with pressure in the fluid domain at 7 mbar of pure CO_2_. For simplicity, the small amounts of water vapor in the fluid parameters were not considered in this thermal simulation. Instead, the one-point measurement of relative humidity from the experimental data (RH_a_) was used, and the value for each grid point of the rest of the chamber was derived using the obtained air temperature, grid point temperature and pressure values from the simulation to calculate the relative humidity using Equations (1)–(5). This helps to visualize the water vapor distribution within a space with large thermal contrasts. When the table is refrigerated, its relative humidity is saturated or oversaturated. As the temperature increases and becomes more similar to the one of the air, the relative humidity of the air increases, and the final result, when the ground and air temperature are closer, is more uniform. The computer simulation is also useful for understanding the thermal equilibration process’ time scale and for comparing it with the actual experimental runs.

The RH_g_^i^ (relative humidity of the ground table with respect to ice) and RH_g_^l^ (relative humidity of the ground table with respect to liquid) are supersaturated as the water from the atmosphere rapidly condensates on the table when the table is cooled using liquid nitrogen. In fact, they maximized at 120% as the frost started to form after 15 min, corresponding to a situation similar to the one in Figure 5a. At 135th min, the RH_g_^l^ peaks at 102% as the liquid water droplets begin to appear on the table, creating a situation similar to the one of Figure 5b. While this water is rapidly released into the air, the RH_a_ of the air increases (in spite of the increasing temperature, which, in the case of a constant amount of water, would have produced a reduction of RH), corresponding to a situation similar to the one of Figure 5c,d.

**Figure 6:**
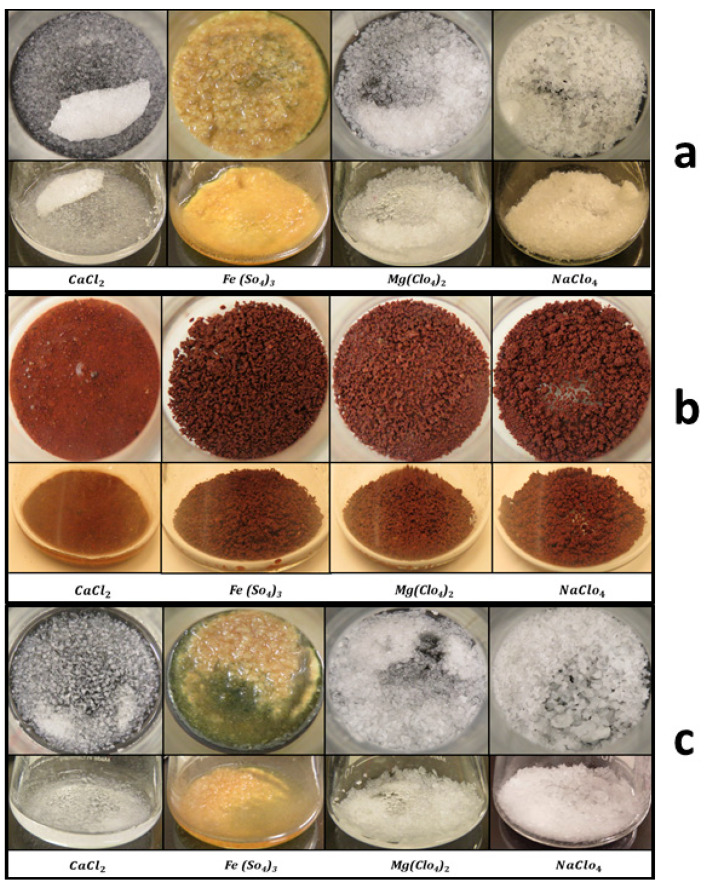
Images of the samples subjected to the simulated Martian atmosphere. (**a**) Experiment 1: The set of images taken after the experiment, which indicates the capture of water from the formation of brines in CaCl_2_ mixture and sponge-like textures in Fe (So_4_)_3_, Mg (ClO_4_)_2_ and NaClO_4_. (**b**) Experiment 2: These are mixtures with 10% salts of the simulant weight showing brine formation in CaCl_2_ and darkening of the simulant observed in the other three mixtures indicating water capture and hydrated state. (**c**) Experiment 3: Same as in (**a**) for different environmental conditions.

As for the salt and SAP mixtures in the beakers, the RH_g_, T_g_, conditions passed well beyond their eutectic temperatures and DRH, allowing for the formation of hydrates of some of them and liquid brines of some others (see Figure 6a). The mixtures of salts and SAP from the beaker were removed and weighed (see Table 1). The amount of water absorbed by them was 3.53 g. After exposure to 6.5 h of evaporation under saturated surface conditions, a total of 53.25 % from the initially injected water (8 g) was still recovered on the surface as pure liquid water or as hydrate or brine.

### 4.2. Experiment 2

**Figure 7:**
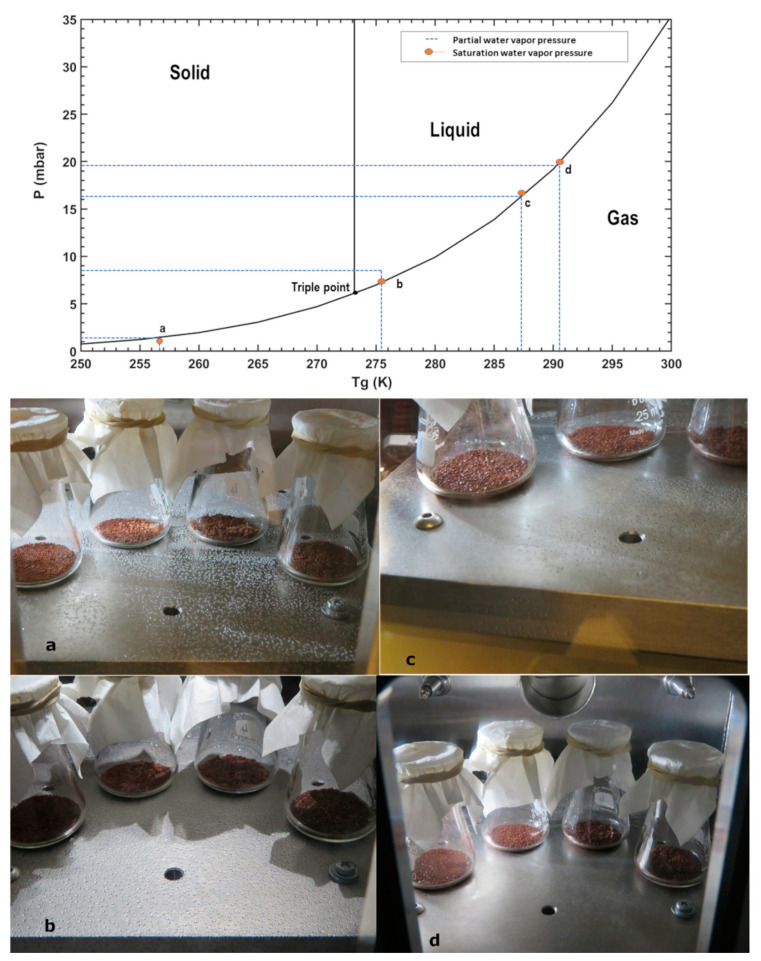
(Top) Environmental P, T_g_ conditions, during this experiment, the surface RHg was 100%, and the air was always drier. (Bottom) Images of the sample taken at different time intervals show the different phase transitions of water droplet formation and evaporation and the mixture of soil simulant with salts. (**a**) When the table temperature T_g_ reached 251 K, the frost started to form. (**b**) As the table temperature T_g_ passed through the triple point at 274 K, the liquid water droplets were formed. (**c**) These droplets were stable till 283 K, and they started evaporation on the sides. (**d**) All the tiny droplets were evaporated at 287 K, whereas some relatively large droplets were extracted through a syringe after the chamber door was open.

This experiment was similar to Experiment 1. However, the beakers had now Mojave Martian Simulant (MMS) mixed with salts to observe changes in the texture of the regolith caused by the interaction with hydrates and liquid brines, and to quantify the potential of the Martian regolith to absorb and retain water. The salts were mixed in four glass beakers with 10% of the simulant weight (1.5 g MMS + 0.15 g salt). After Martian pressure conditions were set, water was injected in increments of 0.5 mL. We started the first water injection as the pressure rose to 7.008 mbar with relative humidity RH_a_ 0.83% and the table temperature T_g_ at 292.3 K. After the last insertion of water, the relative humidity RHa rose to 80.78% with pressure at 7.77 mbar. We injected 14 times in a total of 7 mL of water and maintained the pressure by manipulating the rotary pump valve, releasing the gas seven times. Then, the table was cooled down using LN_2_ as the table temperature T_g_ reached 252 K, the RH_a_ dropped, and frost started to form on the table (Figure 7a) after 4 min, and we stopped the LN_2_ supply. During all the experiments, the surface was supersaturated or saturated. The table temperature T_g_ raised slowly to ambient temperature. As it traversed above the triple point of water, the frost started to turn into water droplets. After 143 min, the first droplet appeared at 7.64 mbar, a relative humidity RH_a_ of 36.31% and a table temperature T_g_ 275.4 K. As the temperature increased, the table was full of liquid water droplets (Figure 7b).

At 340 min, once T_g_ crossed 287.3 K, the droplet started to evaporate from the sides of the table (Figure 7c), at pressure 7.88 mbar and RH_a_ 74.63%. By 500 min of exposure, almost all the small water droplets evaporated from the surface of the table (Figure 7d). Only the larger ones were left when the pressure was 7.92 mbar, RH_a_ 86.105% and T_g_ 290.54 K. The experiment was stopped at this point, and the remaining larger droplets were absorbed with a syringe after opening the chamber door for weighing at ambient lab conditions (Table 1). As for the salt and SAP mixtures in the beakers, the RH, T conditions passed well over their eutectic temperatures and DRH. This allowed the formation of some of the hydrates and liquid brines of some others (see Figure 6b). We recovered 3.15 g of water which is 45% of the mass of injected water. Here the pure liquid water was stable for 4.5 h after passing the triple point of water.

### 4.3. Experiment 3

**Figure 8:**
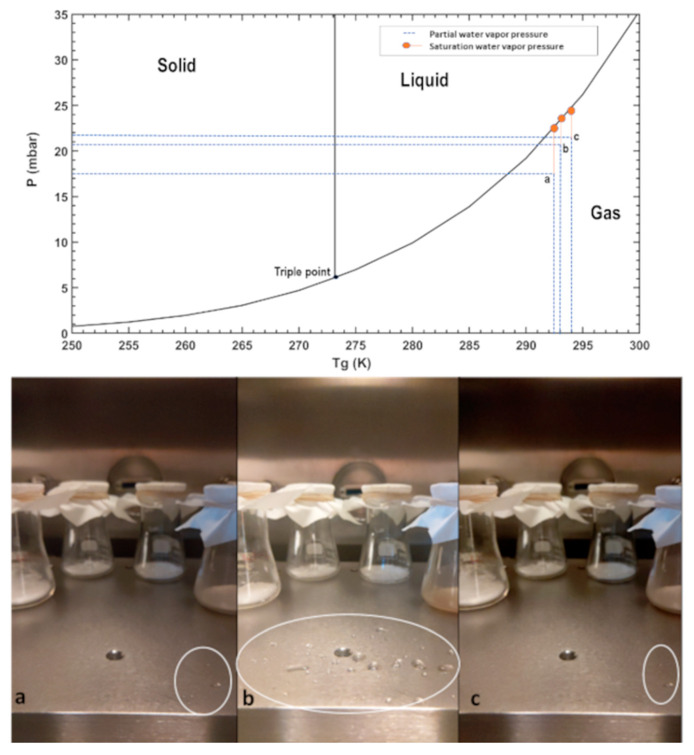
(**Top**) Environmental P, T_g_ conditions, during this experiment, the air and ground were in equilibrium, at almost saturated conditions, and the salts in the beakers were absorbing water. (**Bottom**) Images were taken as the water droplets formed inside the simulated Martian environment on the table. (**a**) As the water is injected at 292.6 K, the first few droplets start to form. (**b**) More droplets begin to form and be stable at 293 K. (**c**) These droplets persist for a period of one hour in the gas phase, and finally, the last droplet evaporated at 294 K.

This experiment was aimed at understanding the evaporation of pure liquid water away from the triple point temperature when both the air and ground are at the same temperature and relative humidity conditions are near saturation. Water was injected in small increments to allow for equilibration. This experiment allows for quantifying a limit to the maximal residence time of dew and the behavior of salts exposed to the same conditions. Here, four sample beakers with salts and SAP mixed in 2:1 ratio (1.5 g salt and 0.75 g SAP) were placed in the chamber. Once the chamber was in Martian pressure conditions, water was injected in small increments of 0.5 mL. The first injection was after 3 min, and the pressure increased to 6.228 mbar with relative humidity RH_a_ rising to 0.4% at table temperature T_g_ of 292.3 K, where the droplets evaporated. At the 43rd min, during the thirteenth injection, stable water droplets started to form on the table (Figure 8a) with pressure at 7.84 mbar, relative humidity RH_a_ at 73.77% and the table temperature T_g_ at 292.65 K. At the next water injection, at 48th min, one can observe how droplets start to form on the table (Figure 8b). We injected 25 times, a total 12.5 g of water, until the liquid water was observed at the 58th min when the conditions inside were 8.04 mbar, at relative humidity RH_a_ 89.91% and the table temperature T_g_ at 293 K. Then we let the water droplets evaporate and observed at what conditions the evaporation started. At the 105th min, only one droplet was on the table (Figure 8c), and it started to evaporate at 8.04 mbar with relative humidity RH_a_ at 93.55% and table temperature T_g_ at 294 K. As the time progressed, the last droplet was evaporated after 108 min with the final pressure conditions at 8.02 mbar, relative humidity RH_a_ at 92.87% and table temperature T_g_ at 294 K. The environmental conditions of the experiment are indicated in Figure 3b.

As for the salt and SAP mixtures in the beakers, the (RH, T) conditions passed well over their eutectic temperatures and DRH, this allowed for the formation of hydrates for some of them and liquid brines for others (see Figure 6c). After weighing the salts, the total water absorbed was 5.42 g. Overall, 43.36% of the injected water was recovered. This is summarized in Table 1. Here we found that the pure liquid water was stable on the working plate for 1 h.

## 5. Discussion

In order to understand the small-scale processes that are involved in the water cycle on Mars and to quantify the existing near-surface water reservoirs for life and exploration, there is a need to perform extensive experimental simulations under controlled laboratory conditions. This is an example of one of these studies, with dedicated sensors, where quantitative conclusions can be obtained despite the inherent limitations of simulating a planetary environment within a small facility. In this study, we have investigated the transient stability of pure liquid water around the triple point and the processes that may induce changes in the water content of the regolith through a set of experiments where we have simulated the environmental conditions on Mars, including pressure, temperature gradients and relative humidity variations. For this purpose, we have conducted three experiments in a Martian chamber, considering the working table as the non-porous Martian bedrock. We have considered a total water column of about 90 pr-µm (or g/cm^2^) to provide sufficient water. The analysis of the data of the REMS instrument on the Curiosity rover shows that there is a drastic reduction in the water content of the atmosphere, suggesting that the soil can absorb up to 15 g/m^2^ within one night, and that this water is released back to the atmosphere during the day. This interchange seems to vary with seasons. Another instrument onboard the Curiosity rover, ChemCam, has also measured during daytime (from 10:00 to 14:00 LTST) the seasonal variations between 2 pr-µm (2 g/m^2^) and 12 pr-µm (12 g/m^2^) [38]. The Thermal Emission Spectrometer (TES) and Mars Global Surveyor observations have demonstrated a maximum in water vapor abundance is observed at high latitudes during midsummer, reaching a maximum value of 100 pr-mm in the northern hemisphere and 50 pr-mm in the southern hemisphere [55]. This pattern was also confirmed by the instrument Spectroscopy for the Investigation of the Characteristics of the Atmosphere of Mars (SPICAM) onboard the Mars Express orbiter [56].

From our experiments, we visually confirmed that saturation is reached at night and that after sunrise, the temperature increases above 273 K, and pure liquid water can be formed on the surface of Mars and be stable for 3.5–4.5 h. Considering that there is no exposure to winds or direct sunlight in our experimental facility, our simulations may represent liquid evaporation conditions in sheltered areas, such as caves, under rocks, or in small-sized regions artificially created within parts of spacecrafts in contact with the ground and atmosphere. The “residence time” or duration of stability, and the amount of water captured, are summarized in Table 1. Experiments 1 and 2 simulate the surface temperature increase from the freezing point to the vicinity of the triple point of water in the saturated surface with respect to water.

Notice that the relative humidity RH is defined as the ratio of water vapor pressure to the saturated water vapor pressure at that temperature, multiplied by 100%. The water vapor pressure is one of the water molecules above the liquid film of water or above the water ice. The saturated water vapor pressure is a function of temperature only and does not depend on the presence of other gases. So, for a given measured or derived RH, one can calculate the partial water vapor pressure at that temperature. These experiments show that when there is a cold sink that traps water, such as the ground at a very low temperature, when the water is released back to the atmosphere, the partial pressure of water just above this reservoir increases freely, even at levels above the total pressure of CO_2_. As the system is out of equilibrium, this large pressure difference should push the water molecules into the upper layers of the atmosphere while the solid ice layer or liquid film would continue to sublimate or evaporate until exhaustion. Moreover, these near-surface water cycle dynamics would be followed by a rapid redistribution of water in the atmospheric column, particularly within the boundary layer, whose temperature evolves during the day. Finally, water is pumped out of the atmosphere through atmospheric escape processes: the hydrogen and oxygen present in these water molecules pushed to higher altitudes could be accountable for the atmospheric escape observations [57,58], which are lost at a rate of 2–3 kg/s to space on present day Mars and even at a higher rate if there is enhanced solar UV radiation.

We have also simulated a plausible near-surface water cycle on Mars to study the atmospheric–regolith interchange using the four salts used in the HABIT instrument to study in situ the water cycle on Mars within the ESA/Roscosmos’ ExoMars 2022 mission. Our experiments mimic the instruments performance on Mars, and the capability of the salts to absorb water as hydrates and then as liquid brines under Martian conditions. The experimental salt mixtures have increased the weight (through water absorption) between 32 and 47% with respect to their initial weight of 9 g, after exposure to about 7.5 h of Martian conditions with atmospheric vapor. This demonstrates that these products are viable substances for water harvesting in present-day Martian conditions. Finally, 45% of the atmospheric water was captured and retained when mixed with MMS dust as regolith emulants.

Interestingly, our experiments show that the regolith could absorb as much as 50% of its weight in water when the surface reaches saturation for a few hours, in this case for a mixture of 10% CaCl_2_ in MMS dust. Furthermore, some granular structures appeared on the mixture of regolith with iron sulfate (see Figure 6b), indicating the cohesive forces of water and brines. The experiments have shown that the metallic table can capture up to 45% of the injected mass of water in the form of the dew of pure liquid water and 0.6 g of salts used in the experiments.

The liquid water evaporation rate, which is roughly derived from Experiment 1, 8 g/(20 cm × 2 cm)/3.5 = 0.057 g/cm^2^/h is in fact comparable to the previous theoretical prediction and experimental observation of 0.055 g/cm^2^/h, at 6.7 mb, by [6].

There are inherent limitations to what can be experimentally tested within a Mars simulation facility. A reduced environment can hardly mimic the large-scale processes on a planet, including the water mixing dynamics over the full length of the planetary boundary layer and the role of global circulation and topographic flows, etc., and cannot simulate the different gravity of Mars. There are large thermal gradients within this space because the chamber is in turn coupled thermally with the external ambient of the laboratory, but these gradients serve to simulate the thermal stratification between the ground and air at night-time.

## 6. Conclusions, Implications, and Future Work

Our experiments confirm that pure liquid water could be formed and be transiently stable under present-day Mars conditions if saturation is reached at the surface. While the actual vapor pressure of water in the atmosphere is less than the saturation vapor pressure of the liquid on the surface, any liquid water will evaporate until either the air is saturated, or the liquid supply is exhausted. These experiments simulate evaporation under wind-free conditions. This scenario is not unrealistic as, according to REMS observations, the night-time to sunrise winds may be very mild with speeds under 2 m/s [59]. Our experiments show how a pool of water is formed and remains stable for about 3.5 to 4.5 h while evaporating and releasing water to the dry atmosphere. This is similar to the process of dew formation and evaporation on Earth. On Earth, dew is composed of liquid water droplets that are formed as water vapor condenses on cold surfaces, mostly at night, on surfaces of temperature below its dew point temperature. This is especially interesting to condense water from the atmosphere and provide a source of water for the survival of individual plants and animals in dryland regions where liquid water is scarce. A recent study [60] about dew formation in a semi-arid plateau in China has shown that the maximum dew residence time was about 18 h/day.

A mixture of 10% of deliquescent salts with the regolith can absorb as much as 18% (for ferric sulfate), or 50% (for calcium chloride), of its weight in water; when exposed to the hydration cycle it forms some granular structures, similar to the ones observed on dunes of Mars. These experiments also suggest that once the water has been captured in the regolith, it can be released later and can produce new chemistry. When exposed to UV, water molecules attached to the regolith may be broken apart by photolysis, leading to the production of oxygen (O_2_) and hydrogen (H_2_), and thus be a source for the recent detection of abnormal seasonal variations of the atmospheric concentration of O_2_ on the surface of Mars [61]. Finally, the existence of this non-equilibrium water interchange between the atmosphere and the regolith may have implications on the formation of avalanches and explain the dry–wet mechanisms [62,63,64] of the downslope movement of materials that are observed on Mars on specific slopes and certain seasons. We conclude that the relative humidity values at night-time on Mars may allow for significant water absorption by the ground, which is released at sunrise. The water cycle dynamics near the surface are therefore always out of equilibrium. In particular, after frost formation, thin films of water may survive for a few hours. This has implications for the present-day habitability of the Martian surface and planetary protection policies.

Future experiments will be focused on controlled experiments to quantify independently every process and to investigate the amount of water absorbed over time by separating each salt into different observations and also to test for the absorption capacity of the MMS-1 simulant without any salts. We will consider further experiments on varying surface conditions to reproduce the diurnal surface variation (including partial water pressure control and thermal profile) expected at different latitudes and seasons, with lower air relative humidity (smaller amounts of water), simulating what happens when the salts are buried within a thicker layer of regolith or mixed in different ratios, or in shadowed regions, such as caves, and to explore the stability of frozen brines.

## Figures and Tables

**Table 1:** Results of the simulations of the water-cycle interaction between the atmosphere and the ground under Martian conditions. Summary of the amount of water captured as weight total percentage (wt%) (mass increase/total weight) in the product, the final increase in mass of water and, for the case of formation of pure liquid water, the residence time, i.e., time of observation of water as liquid beyond the point of stability.

	Experiment 1 (Salt + SAP)			Experiment 2 MMS + Salt)			Experiment 3 (Salt + SAP)		
	wt%	Mass (g)	Residence Time	wt%	Mass (g)	Residence Time	wt%	Mass (g)	Residence Time
Calcium chloride (CaCl_2_)	47%	1.06 g		50%	0.82 g		85%	1.91 g	
Ferric sulphate Fe_2_(SO_4_)_3_	32%	0.71 g		18%	0.3 g		42%	0.95 g	
Magnesium perchlorate Mg (CslO_4_)_2_	41%	0.93 g		36%	0.59 g		68%	1.53 g	
Sodium-perchlorate (NaClO_4_)	37%	0.83 g		30%	0.49 g		46%	1.03 g	
Pure liquid water on the table		0.73 g	3.5 h		0.95 g	4.5 h		0 g	1 h

## Data Availability

They are publicly available upon reasonable request.

## References

[1] Martín-Torres F.J., Zorzano M.-P., Valentín-Serrano P., Harri A.-M., Genzer M., Kemppinen O., Rivera-Valentin E.G., Jun I., Wray J., Bo Madsen M. (2015). Transient Liquid Water and Water Activity at Gale Crater on Mars. Nat. Geosci..

[2] Pollack J.B., Colburn D., Kahn R., Hunter J., Van Camp W., Carlston C.E., Wolf M.R. (1977). Properties of Aerosols in the Martian Atmosphere, as Inferred from Viking Lander Imaging Data. J. Geophys. Res..

[3] Jones K.L., Arvidson R.E., Guinness E.A., Bragg S.L., Wall S.D., Carlston C.E., Pidek D.G. (1979). One Mars Year: Viking Lander Imaging Observations. Science.

[4] Levin G.V., Levin R.L. (1998). Liquid Water and Life on Mars. Instruments, Methods, and Missions for Astrobiology.

[5] Savijärvi H., Paton M., Harri A.-M. (2018). New Column Simulations for the Viking Landers: Winds, Fog, Frost, Adsorption?. Icarus.

[6] Hecht M.H. (2002). Metastability of Liquid Water on Mars. Icarus.

[7] Kuznetz L.H., Gan D.C. (2002). On the Existence and Stability of Liquid Water on the Surface of Mars Today. Astrobiology.

[8] Davy R., Davis J.A., Taylor P.A., Lange C.F., Weng W., Whiteway J., Gunnlaugson H.P. (2010). Initial Analysis of Air Temperature and Related Data from the Phoenix MET Station and Their Use in Estimating Turbulent Heat Fluxes. J. Geophys. Res. Planets.

[9] Morgan P., Smrekar S.E., Lorenz R., Grott M., Kroemer O., Müller N. (2017). Potential Effects of Surface Temperature Variations and Disturbances and Thermal Convection on the Mars InSight HP3 Heat-Flow Determination. Space Sci. Rev..

[10] Ingersoll A.P. (1970). Mars: Occurrence of Liquid Water. Science.

[11] Clark B.C., Van Hart D.C. (1981). The Salts of Mars. Icarus.

[12] Brass G.W. (1980). Stability of Brines on Mars. Icarus.

[13] Haberle R.M., Mckay C.P., Schaeffer J., Cabrol N.A., Grin E.A., Zent A.P., Quinn R. (2001). On the possibility of liquid water on present-day Mars. J. Geophys. Res. Planets.

[14] Chevrier V.F., Altheide T.S. (2008). Low Temperature Aqueous Ferric Sulfate Solutions on the Surface of Mars. Geophys. Res. Lett..

[15] Zorzano M.P., Mateo-Martí E., Prieto-Ballesteros O., Osuna S., Renno N. (2009). Stability of Liquid Saline Water on Present Day Mars. Geophys. Res. Lett..

[16] Gough R.V., Chevrier V.F., Tolbert M.A. (2014). Formation of Aqueous Solutions on Mars via Deliquescence of Chloride-Perchlorate Binary Mixtures. Earth Planet. Sci. Lett..

[17] Rennó N.O., Bos B.J., Catling D., Clark B.C., Drube L., Fisher D., Goetz W., Hviid S.F., Keller H.U., Kok J.F. (2009). Possible Physical and Thermodynamical Evidence for Liquid Water at the Phoenix Landing Site. J. Geophys. Res. E Planets.

[18] Nuding D.L., Davis R.D., Gough R.V., Tolbert M.A. (2015). The Aqueous Stability of a Mars Salt Analog: Instant Mars. J. Geophys. Res. Planets.

[19] Fischer E., Martínez G.M., Renn N.O. (2016). Formation and Persistence of Brine on Mars: Experimental Simulations throughout the Diurnal Cycle at the Phoenix Landing Site. Astrobiology.

[20] Heinz J., Schulze-Makuch D., Kounaves S.P. (2016). Deliquescence-Induced Wetting and RSL-like Darkening of a Mars Analogue Soil Containing Various Perchlorate and Chloride Salts. Geophys. Res. Lett..

[21] Nikolakakos G., Whiteway J.A. (2018). Laboratory Study of Adsorption and Deliquescence on the Surface of Mars. Icarus.

[22] Primm K.M., Gough R.V., Wong J., Rivera-Valentin E.G., Martinez G.M., Hogancamp J.V., Archer P.D., Ming D.W., Tolbert M.A. (2018). The Effect of Mars-Relevant Soil Analogs on the Water Uptake of Magnesium Perchlorate and Implications for the Near-Surface of Mars. J. Geophys. Res. Planets.

[23] Martínez G.M., Renno N.O. (2013). Water and Brines on Mars: Current Evidence and Implications for MSL. Space Sci. Rev..

[24] Davila A.F., Duport L.G., Melchiorri R., Jänchen J., Valea S., De Los Rios A., Fairén A.G., Möhlmann D., McKay C.P., Ascaso C. (2010). Hygroscopic Salts and the Potential for Life on Mars. Astrobiology.

[25] McEwen A.S., Ojha L., Dundas C.M., Mattson S.S., Byrne S., Wray J.J., Cull S.C., Murchie S.L., Thomas N., Gulick V.C. (2011). Seasonal Flows on Warm Martian Slopes. Science.

[26] Rivera-Valentín E.G., Chevrier V.F., Soto A., Martínez G. (2020). Distribution and Habitability of (Meta)Stable Brines on Present-Day Mars. Nat. Astron..

[27] Henderson J.A., Lucas J.S. (1971). Adsorption on the Martian Regolith. Nature.

[28] Fanale F.P., Cannon W.A. (1974). Exchange of Adsorbed H_2_O and CO_2_ between the Regolith and Atmosphere of Mars Caused by Changes in Surface Insolation. J. Geophys. Res..

[29] Jakosky B.M., Zent A.P., Zurek R.W. (1997). The Mars Water Cycle: Determining the Role of Exchange with the Regolith. Icarus.

[30] Schorghofer N., Aharonson O. (2005). Stability and Exchange of Subsurface Ice on Mars. J. Geophys. Res. E Planets.

[31] Bryson K.L., Chevrier V., Sears D.W.G., Ulrich R. (2008). Stability of Ice on Mars and the Water Vapor Diurnal Cycle: Experimental Study of the Sublimation of Ice through a Fine-Grained Basaltic Regolith. Icarus.

[32] Chevrier V., Ostrowski D.R., Sears D.W.G. (2008). Experimental Study of the Sublimation of Ice through an Unconsolidated Clay Layer: Implications for the Stability of Ice on Mars and the Possible Diurnal Variations in Atmospheric Water. Icarus.

[33] Meslin P.-Y., Gasnault O., Forni O., Schröder S., Cousin A., Berger G., Clegg S.M., Lasue J., Maurice S., Sautter V. (2013). Soil Diversity and Hydration at Gale Crater, Mars. Science.

[34] Massé M., Conway S.J., Gargani J., Patel M.R., Pasquon K., McEwen A., Carpy S., Chevrier V., Balme M.R., Ojha L. (2016). Transport Processes Induced by Metastable Boiling Water under Martian Surface Conditions. Nat. Geosci..

[35] Chittenden J.D., Chevrier V., Roe L.A., Bryson K., Pilgrim R., Sears D.W.G. (2008). Experimental Study of the Effect of Wind on the Stability of Water Ice on Mars. Icarus.

[36] Martín-Torres J., Zorzano M.-P., Soria-Salinas Á., Nazarious M.I., Konatham S., Mathanlal T., Ramachandran A.V., Ramírez-Luque J.-A., Mantas-Nakhai R. (2020). The HABIT (HabitAbility: Brine Irradiation and Temperature) Environmental Instrument for the ExoMars 2022 Surface Platform. Planet. Space Sci..

[37] Rummel J.D., Beaty D.W., Jones M.A., Bakermans C., Barlow N.G., Boston P.J., Chevrier V.F., Clark B.C., de Vera J.-P.P., Gough R.V. (2014). A New Analysis of Mars “Special Regions”: Findings of the Second MEPAG Special Regions Science Analysis Group (SR-SAG2). Astrobiology.

[38] McConnochie T.H., Smith M.D., Wolff M.J., Bender S., Lemmon M., Wiens R.C., Maurice S., Gasnault O., Lasue J., Meslin P.-Y. (2018). Retrieval of Water Vapor Column Abundance and Aerosol Properties from ChemCam Passive Sky Spectroscopy. Icarus.

[39] Harri A.-M., Genzer M., Kemppinen O., Gomez-Elvira J., Haberle R., Polkko J., Savijärvi H., Rennó N., Rodriguez-Manfredi J.A., Schmidt W. (2014). Mars Science Laboratory Relative Humidity Observations: Initial Results. J. Geophys. Res. Planets.

[40] Sobrado J.M. (2020). Mimicking the Martian Hydrological Cycle: A Set-Up to Introduce Liquid Water in Vacuum. Sensors.

[41] Wu Z., Ling Z., Zhang J., Fu X., Liu C., Xin Y., Li B., Qiao L. (2021). A Mars Environment Chamber Coupled with Multiple In Situ Spectral Sensors for Mars Exploration. Sensors.

[42] Vakkada Ramachandran A., Nazarious M.I., Mathanlal T., Zorzano M.-P., Martín-Torres J. (2020). Space Environmental Chamber for Planetary Studies. Sensors.

[43] Nazarious M.I., AU-Ramachandran A.V., Zorzano M.P., Martin-Torres J. (2021). Measuring Electrical Conductivity to Study the Formation of Brines Under Martian Conditions. JoVE.

[44] Shirke Y.M., Abou-Elanwar A.M., Choi W.K., Lee H., Hong S.U., Lee H.K., Jeon J.D. (2019). Influence of Nitrogen/Phosphorus-Doped Carbon Dots on Polyamide Thin Film Membranes for Water Vapor/N2 Mixture Gas Separation. RSC Adv..

[45] Allen C.C., Jager K.M., Morris R.V., Lindstrom D.J., Lindstrom M.M., Lockwood J.P. (1998). JSC MARS-1: A Martian Soil Simulant.

[46] Nørnberg P., Gunnlaugsson H.P., Merrison J.P., Vendelboe A.L. (2009). Salten Skov I: A Martian Magnetic Dust Analogue. Planet. Space Sci..

[47] Gouache T.P., Patel N., Brunskill C., Scott G.P., Saaj C.M., Matthews M., Cui L. (2011). Soil Simulant Sourcing for the ExoMars Rover Testbed. Planet. Space Sci..

[48] Zeng X., Li X., Wang S., Li S., Spring N., Tang H., Li Y., Feng J. (2015). JMSS-1: A New Martian Soil Simulant. Earth Planets Space.

[49] Cannon K.M., Britt D.T., Smith T.M., Fritsche R.F., Batcheldor D. (2019). Mars Global Simulant MGS-1: A Rocknest-Based Open Standard for Basaltic Martian Regolith Simulants. Icarus.

[50] Ladino L.A., Abbatt J.P.D. (2013). Laboratory Investigation of Martian Water Ice Cloud Formation Using Dust Aerosol Simulants. J. Geophys. Res. Planets.

[51] Nazarious M.I., Ramachandran A.V., Zorzano M.-P., Martin-Torres J. (2019). Calibration and Preliminary Tests of the Brine Observation Transition To Liquid Experiment on HABIT/ExoMars 2020 for Demonstration of Liquid Water Stability on Mars. Acta Astronaut..

[52] Martínez G.M., Rennó N., Fischer E., Borlina C.S., Hallet B., de la Torre Juárez M., Vasavada A.R., Ramos M., Hamilton V., Gomez-Elvira J. (2014). Surface Energy Budget and Thermal Inertia at Gale Crater: Calculations from Ground-Based Measurements. J. Geophys. Res. Planets.

[53] Altheide T., Chevrier V., Nicholson C., Denson J. (2009). Experimental Investigation of the Stability and Evaporation of Sulfate and Chloride Brines on Mars. Earth Planet. Sci. Lett..

[54] Vakkada Ramachandran A., Zorzano M.-P., Martin-Torres J. (2021). Numerical Heat Transfer Study of a Space Environmental Testing Facility Using COMSOL Multiphysics. Therm. Sci. Eng. Prog..

[55] Smith M.D. (2002). The Annual Cycle of Water Vapor on Mars as Observed by the Thermal Emission Spectrometer. J. Geophys. Res. Planets.

[56] Fedorova A., Korablev O., Bertaux J.-L., Rodin A., Kiselev A., Perrier S. (2006). Mars Water Vapor Abundance from SPICAM IR Spectrometer: Seasonal and Geographic Distributions. J. Geophys. Res. Planets.

[57] Jakosky B.M., Brain D., Chaffin M., Curry S., Deighan J., Grebowsky J., Halekas J., Leblanc F., Lillis R., Luhmann J.G. (2018). Loss of the Martian Atmosphere to Space: Present-Day Loss Rates Determined from MAVEN Observations and Integrated Loss through Time. Icarus.

[58] Dong C., Lee Y., Ma Y., Lingam M., Bougher S., Luhmann J., Curry S., Toth G., Nagy A., Tenishev V. (2018). Modeling Martian Atmospheric Losses over Time: Implications for Exoplanetary Climate Evolution and Habitability. Astrophys. J..

[59] Newman C.E., Gómez-Elvira J., Marin M., Navarro S., Torres J., Richardson M.I., Battalio J.M., Guzewich S.D., Sullivan R., Torre M. (2017). Winds Measured by the Rover Environmental Monitoring Station (REMS) during the Mars Science Laboratory (MSL) Rover’s Bagnold Dunes Campaign and Comparison with Numerical Modeling Using MarsWRF. Icarus.

[60] Jia Z., Wang Z., Wang H. (2019). Characteristics of Dew Formation in the Semi-Arid Loess Plateau of Central Shaanxi Province, China. Water.

[61] Trainer M.G., Wong M.H., McConnochie T.H., Franz H.B., Atreya S.K., Conrad P.G., Lefèvre F., Mahaffy P.R., Malespin C.A., Manning H.L.K. (2019). Seasonal Variations in Atmospheric Composition as Measured in Gale Crater, Mars. J. Geophys. Res. Planets.

[62] Bhardwaj A., Sam L., Martín-Torres F.J., Zorzano M.-P. (2019). Are Slope Streaks Indicative of Global-Scale Aqueous Processes on Contemporary Mars?. Rev. Geophys..

[63] Bhardwaj A., Sam L., Martín-Torres F.J., Zorzano M.P. (2019). Discovery of Recurring Slope Lineae Candidates in Mawrth Vallis, Mars. Sci. Rep..

[64] Tebolt M., Levy J., Goudge T. (2019). Slope, elevation, and thermal inertia trends of martian recurring slope lineae initiation and termination points: Multiple possible processes occurring on coarse, sandy slopes. Icarus.

